# *De novo* genome assembly of a native *Saccharomyces cerevisiae* strain isolated from spontaneous fermentation of vineyard must in the Atacama Desert (Chile)

**DOI:** 10.1093/femsyr/foag033

**Published:** 2026-08-04

**Authors:** Héctor Aguayo-Cumplido, Michelle Cifras-Céspedes, Marcelo Lanino, Ingrid Poblete, Jennifer Molinet, Lía Ramírez-Fernández

**Affiliations:** Laboratorio de Genómica de Ambientes Extremos, Facultad de Recursos Naturales Renovables, Universidad Arturo Prat, Iquique 1110939, Chile; Núcleo de Investigación Aplicada e Innovación en Ciencias Biológicas, Facultad de Recursos Naturales Renovables, Universidad Arturo Prat, Iquique 1110939, Chile; Laboratorio de Genómica de Ambientes Extremos, Facultad de Recursos Naturales Renovables, Universidad Arturo Prat, Iquique 1110939, Chile; Núcleo de Investigación Aplicada e Innovación en Ciencias Biológicas, Facultad de Recursos Naturales Renovables, Universidad Arturo Prat, Iquique 1110939, Chile; Núcleo de Investigación Aplicada e Innovación en Ciencias Biológicas, Facultad de Recursos Naturales Renovables, Universidad Arturo Prat, Iquique 1110939, Chile; Núcleo de Investigación Aplicada e Innovación en Ciencias Biológicas, Facultad de Recursos Naturales Renovables, Universidad Arturo Prat, Iquique 1110939, Chile; Facultad de Ingeniería, Instituto de Ciencias Aplicadas, Centro de Investigación e Innovación, Universidad Autónoma de Chile, Santiago 8581151, Chile; Laboratorio de Genómica de Ambientes Extremos, Facultad de Recursos Naturales Renovables, Universidad Arturo Prat, Iquique 1110939, Chile; Núcleo de Investigación Aplicada e Innovación en Ciencias Biológicas, Facultad de Recursos Naturales Renovables, Universidad Arturo Prat, Iquique 1110939, Chile

**Keywords:** *Saccharomyces cerevisiae*, Atacama Desert, hybrid genome assembly, osmotic stress, thermal tolerance, native yeast

## Abstract

In this work, we investigated the genomic and phenotypic basis of stress tolerance in the native *Saccharomyces cerevisiae* strain M6, isolated from spontaneously fermenting must at a vineyard located in Atacama Desert, through a *de novo* hybrid genome assembly generated using short- and long-read sequencing technologies. The assembly comprised 11.89 Mb with high completeness (99.5%), and flow cytometry analysis confirmed a diploid genome organization. Phylogenomic analyses placed M6 within the Wine/European lineage, although displaying genomic divergence relative to other wine-associated strains. Using S288C as the reference genome, comparative variant analysis was performed for M6 and four closely related strains, including Wine/European and Alpechin strains. Coding variants were identified in genes associated with osmotic sensing and signaling (*SSK1, SSK2*), trehalose metabolism (*TPS2, NTH1*). Growth assays demonstrate that M6 exhibits enhanced performance under elevated temperatures (35°C–38°C) and high salinity (up to 1.5 M NaCl) compared with commercial and laboratory strains. In addition, Biolog YT assays revealed broad carbohydrate utilization capacity, including the metabolism of maltose, galactose, and raffinose-family oligosaccharides. Together, these results provide integrated genomic and phenotypic evidence of stress resistance and metabolic flexibility in strain M6, highlighting its potential as a biotechnological resource for fermentation processes

## Teaser

A desert wine yeast showed enhanced tolerance to heat and salt, revealing genetic and metabolic traits with potential applications in industrial fermentation.

## Introduction

*Saccharomyces cerevisiae* is a model eukaryotic organism of fundamental importance for bread, beer, and wine production, and it was the first eukaryote to have its genome fully sequenced (Goffeau et al. 1996). However, although large-scale population genomic studies have substantially expanded our understanding of natural and industrial diversity (Gallone et al. 2016, Peter et al. 2018, Loegler et al. 2024), strains originating from extreme and underexplored environments remain comparatively underrepresented in detailed genomic, physiological, and metabolic analyses.

Environmental strains provide a valuable window into the natural diversity and adaptive potential of *S. cerevisiae* (Peter et al. 2018). Yeast isolates from spontaneous fermentations as well as from natural ecosystems are exposed to multiple environmental stresses absent from controlled fermentations, including osmotic stress, thermal stress, heavy metal toxicity, nutrient limitation, oxidative stress, and microbial competition (Fleet 2003, Mukherjee et al. 2017, Molinet and Stelkens 2025), often harboring unique genetic traits linked to stress tolerance and niche adaptation (Molinet and Cubillos 2020, Molinet et al. 2022, Guedes et al. 2024). The study of such strains can reveal evolutionary mechanisms and genetic determinants with significant biotechnological relevance. These may include variation in pathways related to stress response, detoxification, and nutrient metabolism (Segal-Kischinevzky et al. 2022), as well as mechanisms such as modulation of membrane lipid composition to maintain fluidity (Henderson et al. 2013), as described in thermo- and osmotolerant yeast species (Buzzini et al. 2018).

The Atacama Desert in northern Chile represents an exceptional natural laboratory for studying microbial adaptation. As the driest non-polar desert on Earth and a global hotspot for ultraviolet radiation (Lehner et al. 2001), it hosts small-scale viticultural systems that generate fermentative environments exposed to combined stresses, including high salinity, strong thermal fluctuations, and intense radiation (Poblete et al. 2020, Lanino and Poblete 2022). These conditions create a unique selective landscape for yeasts thriving in spontaneous fermentations.

More broadly, fermentative environments associated with winemaking are increasingly challenged by elevated sugar concentrations and higher fermentation temperatures as a consequence of global warming (van Leeuwen and Darriet 2016). In addition to these changes, grape must composition is also affected, including shifts in nitrogen and vitamin availability, as well as in organic acid profiles and pH. Together, these factors favor *S. cerevisiae* and non-*Saccharomyces* strains capable of maintaining metabolic performance and cellular integrity under osmotic, ethanol, and pH stress conditions (García et al. 2016). Accordingly, indigenous yeast populations adapted to local environments have emerged as powerful models for studying robustness and stress tolerance in natural fermentative niches (Petrignani et al. 2024, Moreira-Ramos et al. 2024, Flores et al. 2025).

Native yeast populations can persist across vintages and fermentation stages, reflecting stable ecological associations with specific vineyards and wineries (Börlin et al. 2020). This persistence underpins the concept of microbial terroir, whereby locally adapted microbial communities contribute to the regional identity and aromatic profile of wines (Gilbert et al. 2014, Carrau et al. 2020, Teixeira et al. 2024). The identification and preservation of such native yeasts is therefore essential for understanding both ecological processes and fermentation outcomes.

Despite the extreme environmental conditions of northern Chile, no *S. cerevisiae* strains from this region have been genomically characterized to date. The native strain M6, isolated from spontaneous fermentations in the hyper-arid Atacama Desert, therefore represents the first genomic insight into *S. cerevisiae* isolated from this ecosystem.

In this study, we present a *de novo* hybrid genome assembly and functional annotation of the native *S. cerevisiae* strain M6. Genome assembly was performed *de novo* using a hybrid strategy that integrates long Nanopore reads to maximize assembly continuity and short high-quality reads to improve base-level accuracy, without the use of a reference genome (Goodwin et al. 2015). We characterize its genome structure, ploidy, phylogenetic placement, and carbon-source utilization profile, and identify genes and sequence variants potentially associated with stress resilience and metabolic flexibility. Together, our results advance the understanding of yeast adaptation to extreme fermentative environments and highlight the biotechnological potential of native strains for improving fermentation performance under challenging conditions.

## Methods

### Sampling site

The M6 yeast strain was isolated from spontaneously fermenting must obtained from a *Vitis vinifera* cv. Tamarugal grapevine, cultivated in the Canchones Vineyard, located in the Pampa del Tamarugal (Atacama Desert, Chile). This hyper-arid region lies within the intermediate depression of northern Chile and represents a site with a long-standing viticultural tradition in the Tarapacá region. The vineyard is part of the Canchones Experimental Center of the Universidad Arturo Prat (20°26′ S, 69°32′ W). At the time of harvest, the must had a soluble solids content of 22.4 °Brix and a pH of 3.5, and was obtained after pressing and subsequently maintained in stainless steel tanks under standard vinification conditions. This area is characterized by an arid climate, large temperature fluctuations, with absolute maximum temperatures exceeding 34°C throughout the year and reaching historical extremes of up to 42°C, while average minimum temperatures are around 8.2°C and absolute minima can drop to −8.6°C and high levels of UV radiation (Lehner et al. 2001, Poblete et al. 2020, Lanino and Poblete 2022).

### Isolation and molecular characterization of yeast strain M6

Approximately 5 g of fermented grape must were placed into 15 ml Falcon tubes containing 5 ml of sterile distilled water. The tubes were then agitated for 5 min at maximum speed using a WiseMix® shaker. After agitation, 100 µl of fermented grape mash suspension was inoculated onto yeast growth solid medium (YEPD: 2% agar, 1% yeast extract, 2% peptone, 2% glucose). Inoculation was performed by streaking with a sterile glass loop. The plates were incubated at room temperature for two days or until visible colony growth was observed. All procedures were carried out under a laminar flow hood.

Yeast colonies were examined under an OLYMPUS BX53 microscope at 100 × magnification to identify morphological features characteristic of yeasts, including cell size, budding patterns, and the presence of ascospores, in order to confirm yeast identity and distinguish them from potential bacterial contaminants.

A total of 10 yeast isolates were obtained from the must sample. Colonies presumptively identified as *S. cerevisiae* were confirmed by colony PCR amplification using *S. cerevisiae* species-specific primers ScerF2 (5′-GCGCTTTACATTCAGATCCCGAG-3′) and ScerR2 (5′-TAAGTTGGTTGTCAGCAAGATTG-3′). PCR reactions were carried out under standard conditions, and amplification products were verified by agarose gel electrophoresis. Selected colonies were preserved in glycerol stocks at –80°C for long-term storage.

A preliminary phenotypic screening was then performed to evaluate thermal and osmotic stress tolerance among the *S. cerevisiae* isolates. Among them, strain M6 exhibited the highest tolerance under both conditions and was therefore selected for subsequent molecular, genomic, and functional analyses.

For the molecular identification of the M6 strain, the internal transcribed spacer (ITS) region was amplified using primers ITS4 (5′- TCCTCCGCTTATTGATATGC -3′) and ITS5 (5′- GGAAGTAAAAGTCGTAACAAGG -3′). The amplicon was sequenced in both forward and reverse directions, and capillary electrophoresis sequencing was performed on an Applied Biosystems 3500 Genetic Analyzer at the Austral-omics Core Facility. Raw sequences were manually cleaned and curated using the APE v.5 (Paradis et al. 2019), and the resulting consensus sequence was compared against the NCBI nucleotide (nt) database using the BLASTn tool (Altschul et al. 1990)

A phylogenetic tree was constructed using the closest ITS sequences retrieved from the NCBI database (Sayers et al. 2020). Sequences were first aligned using MUSCLE implemented in MEGA (Tamura et al. 2007), followed by the construction of a maximum likelihood phylogenetic tree with 1 000 bootstrap replicates, also performed in MEGA. The resulting tree was visualized and edited using the Interactive Tree of Life (iTOL) online tool (https://itol.embl.de).

### Whole-genome sequencing and quality control

High-molecular-weight genomic DNA from the M6 strain was extracted using the GeneJET Genomic DNA Purification Kit (ThermoFisher) following enzymatic lysis with lysozyme and proteinase K. DNA concentration was quantified using Qubit fluorometry (Qubit dsDNA HS Assay), and integrity was verified by 1.5% agarose gel electrophoresis.

For short-read sequencing, 200 ng of DNA was used to prepare libraries with the MGI Easy FS DNA Library Prep Set, and sequencing was performed on the DNBSeq G400 platform, generating 150 bp paired-end reads. For long-read sequencing, 1 μg of DNA was processed using the Oxford Nanopore Native Barcoding 24 V14 kit and sequenced on a MinION flow cell. Basecalling was conducted using DORADO v0.5.1 in high-accuracy mode.

Short-read quality control was conducted using FastQC and MultiQC. Reads were filtered with Trimmomatic (v0.39) (Bolger et al. 2014) and PRINSEQ (v0.20.4) (Cantu et al. 2019) to remove adapters, low-quality bases (Phred < Q30), ambiguous nucleotides, and low-complexity reads. Only sequences with an average Phred score ≥ Q30 were retained for downstream analyses. Long-read quality control was performed with NanoPlot. A Q10 threshold was applied during initial filtering and reads shorter than 10 000 bp were removed using Filtlong. Filtered reads were then concatenated for subsequent genome assembly.

### Genome assembly

Genome assembly was performed using a hybrid polishing strategy involving both long and short reads to enhance sequence accuracy, particularly in repetitive regions or those affected by indels typical of long-read technologies. In the first stage, a baseline assembly was generated using Flye (v2.8.3) (Kolmogorov et al. 2019) with the parameter –nano-raw, based on filtered long reads. This was followed by a first round of polishing using Medaka (v2.0.0), which applies a neural network-based approach to correct systematic Nanopore sequencing errors (https://github.com/nanoporetech/medaka). In the final stage, a second round of polishing was performed using filtered short reads with Pilon (v1.23) (Walker et al. 2014), further improving the consensus sequence.

Assembly quality was assessed using QUAST (v5.0.2) (Gurevich et al. 2013), which provides general quality and completeness metrics. Additionally, assembly completeness was evaluated using BUSCO (v5.6.1) (Simão et al. 2015), employing the Saccharomycetes-specific dataset of single-copy orthologs. The raw data and assembled *de novo* genome have been submitted to the European Nucleotide Archive (ENA) under the accession number PRJEB96299.

### Gene prediction

Structural gene annotation was conducted in two sequential steps. First, GeneMark-ES (v4.38) (Lomsadze et al. 2005) was used with default parameters to generate an ab initio gene prediction, which does not require a reference model and helps reduce bias. Subsequently, AUGUSTUS v3.5.0 (Stanke et al. 2006) was applied to refine gene predictions using Saccharomycetes-specific models, resulting in more accurate annotations aligned with yeast genomic features.

### Average nucleotide identity (ANI)

To assess the genetic relatedness of the M6 genome to known *S. cerevisiae* strains, an Average Nucleotide Identity (ANI) analysis was performed using the OrthoANI algorithm implemented in the OAT software (Lee et al. 2016). The final polished genome assembly of M6 was compared to the reference genome of *S. cerevisiae* S288C (accession: GCA_000146045.2). Default parameters were used, and the resulting ANI value was interpreted to evaluate species-level similarity.

### Ploidy determination

Genome-wide sequencing coverage was calculated from the alignment of strain M6 reads against the *S. cerevisiae* EC1118 reference genome using SAMtools depth. Coverage values were summarized in 10 kb genomic windows, and mean coverage was calculated for each window. To facilitate comparison across the genome, coverage was normalized by dividing each window value by the genome-wide mean coverage, generating relative coverage profiles. A cumulative genomic coordinate system was constructed by concatenating reference contigs, enabling visualization of the entire genome as a single linear representation. Normalized coverage profiles were visualized in R using ggplot2 to assess potential chromosome-scale copy number variation.

To further evaluate ploidy-associated patterns, allele balance was calculated for heterozygous SNPs with sequencing depth ≥ 20 reads (DP ≥ 20). Allele balance was defined as the proportion of reads supporting the alternative allele [ALT/(REF + ALT)] and was estimated using 8870 heterozygous SNPs identified in the M6 genome after quality filtering. Allele balance values were grouped into frequency bins to assess their genome-wide distribution.

In addition, ploidy level was independently estimated using variant-based approaches. High-quality short reads were aligned to M6 *de novo* genome assembly using BWA-MEM (v0.7.18) with default settings. The alignments were processed with SAMtools (v1.21) (Li et al. 2009) and FreeBayes (v1.3.9) to identify single nucleotide polymorphisms (SNPs). Variant calls were filtered for high confidence (Phred score ≥ 30; depth ≥ 10) using VCFtools (v0.1.16) (Danecek et al. 2011). The proportion of heterozygous SNPs (0/1 genotype) was then quantified to infer ploidy, applying a Hardy-Weinberg equilibrium test and extracting heterozygous sites using AWK.

Also, ploidy was assessed through the propidium iodide (PI) staining assay, as previously described (Molinet et al. 2024). Initially, one colony was grown overnight at 25°C on YPD. Then, 1 ml of each culture was harvested and suspended in 2.3 ml of absolute ethanol for fixation at 4°C for 24 h. Following fixation, cells were washed with sodium citrate buffer (50 mM, pH 7), and 100 µl of cells was resuspended in the same solution, with 1 µl of RNase A (100 mg/ml) added, and incubated for 2 h at 37°C. Then, cells were stained with a solution containing PI (50 µg/ml) and sodium citrate (50 mM, pH 7) and incubated for 40 min at room temperature in the dark. Analysis was conducted on a BD FACSCanto II flow cytometer with excitation at 488 nm and fluorescence detection using an FL2-A filter, analyzing 10 000 cells per sample. Two strains with known ploidy (*S. cerevisiae* 2n and *S. pastorianus* 4n) were employed as controls.

### SNP identification and functional annotation

SNPs were identified by aligning the genome of strain M6 against the *S. cerevisiae* reference genome (S288C, GCA_000146045.2) using the MUMmer4 toolkit (Marçais et al. 2018). The alignment was generated with nucmer, and SNPs were extracted using the show-snps utility. Variant coordinates were converted to VCF format using a custom Python script (my-mummer-2-vcf.py), and SNPs were functionally annotated with BCFtools csq (Danecek et al. 2021), using the reference FASTA and GFF3 files.

To identify genes affected by SNPs and determine their involvement in metabolic pathways, gene names were extracted from the BCSQ field using BCFtools query and processed to match KEGG identifiers. Due to discrepancies between common gene names and KEGG IDs, gene names were mapped using the UniProt ID mapping tool (The UniProt Consortium 2023), and a custom conversion file was generated. KEGG pathway annotations were retrieved programmatically via the KEGG REST API (Kanehisa et al. 2016).

To further contextualize the genomic background of strain M6, publicly available whole-genome sequencing datasets representing closest wine-associated and Alpechin *S. cerevisiae* lineages were retrieved from the Sequence Read Archive (SRA). Raw Illumina reads from strains EC1118, DBVPG3051 (CBH), CBS2909 (AID), and YJM1078 (YDM) were quality filtered using fastp v1.3.3. Adapter sequences were automatically detected and removed, bases with Phred quality scores below 20 were filtered, reads were trimmed using a sliding-window mean quality threshold of 25, and reads shorter than 50 bp after trimming were discarded.

Filtered reads were aligned against the *S. cerevisiae* S288C reference genome (GCA_000146045.2) using BWA-MEM. Alignments were sorted and indexed with SAMtools. Joint variant calling was performed using BCFtools v1.23.1 and HTSlib v1.23.1 with BCFtools mpileup and BCFtools call (Danecek et al. 2021). Variants unique to strain M6 were identified by selecting positions carrying an alternative allele in M6 while retaining the reference allele in all comparator strains. Functional effects of variants were predicted using BCFtools csq with the S288C reference genome and corresponding GFF3 annotation. Predicted effects were classified as synonymous, missense, frameshift, stop-gained, stop-lost, start-lost and other functional categories.

To evaluate the genomic relationship between M6 and previously characterized *S. cerevisiae* lineages, a SNP-based clustering analysis was performed. A genotype matrix was generated from the multisample VCF using PLINK v1.9.0-b.8 (Chang et al. 2015), and pairwise genetic distances were calculated using the identity-by-state (IBS) metric. A Neighbor-Joining tree was subsequently constructed from the resulting distance matrix using the ape package in R.

### Phylogenetic placement and divergence analysis of the M6 strain

To investigate the phylogenetic position of the M6 strain, a subset of 67 coding sequences (CDS) was extracted from the genome dataset of 1011 *S. cerevisiae* strains published by Peter et al. (2018). These loci were selected based on the following criteria: (i) presence in all compared genomes, (ii) evidence of single-copy orthology, (iii) absence of ambiguous paralogy, and (iv) alignment quality sufficient for reliable concatenation and phylogenetic inference. The selected genes, listed in Supplementary Table 1, were aligned individually using MAFFT v7.525 (Katoh and Standley 2013). These alignments were then concatenated into a single supermatrix using AMAS v1.0 (Borowiec 2016).

A maximum-likelihood phylogenetic tree was inferred using IQ-TREE version 2.4.0 (Minh et al. 2020), employing the GTR + R substitution model. Branch support was assessed using 1000 ultrafast bootstrap replicates with the -B 1000 option (Hoang et al. 2018).

Pairwise genetic distances were extracted from the resulting cophenetic matrix, allowing comparative analysis of the average divergence of M6 with respect to the full dataset, the subset of Chilean strains, the highly divergent Taiwanese lineage, and its closest phylogenetic neighbor (ABP).

### Chromosomal reordering and detection of extrachromosomal elements

The initial *de novo* assembly was reordered using RagTag v2.1.0 (Alonge et al. 2022) to scaffold and orient contigs based on the *S. cerevisiae* S288C reference genome (NCBI RefSeq: GCA_000146045.2).

Following reordering, the resulting ragtag.scaffold.fasta file was manually inspected. Contigs that did not align to the chromosomes were retained as unplaced sequences.

Coding sequences within non-aligned contigs were predicted using AUGUSTUS v3.5.0 (Stanke et al. 2006), followed by comparative analysis against the *S. cerevisiae* pangenome (Peter et al. 2018). Gene identities were subsequently verified using BLASTn (Altschul et al. 1990).

To identify conserved motifs and functional domains, the online platform InterProScan (Jones et al. 2014) was used. In addition, functional annotation was complemented using eggNOG-mapper v2 (Cantalapiedra et al. 2021).

Specifically, for gene 16 of contig 36, identified as an *RTG1*-like transcriptional regulator, subcellular localization was predicted using DeepLoc v1.0 (Almagro Armenteros et al. 2017). The input consisted of the amino acid sequence of CDS_16, extracted from the *de novo* genome assembly and annotated with InterProScan. The sequence was provided in FASTA format, and default settings were used for the analysis.

### Growth rate assays

Growth kinetics were assessed using a Synergy H1X multi-mode plate reader (BioTek Instruments, USA). Yeast strains included the native *S. cerevisiae* M6, additional *S. cerevisiae* isolates obtained from wine must, the commercial wine strain EC1118 (Lallemand Inc., Canada), and the laboratory reference strain S288C. Cultures were adjusted to an initial optical density (OD600) of ∼0.2 in 200 µl of YPD medium and inoculated into sterile 96-well microplates. Negative controls, containing only YPD medium, were included on each plate. For thermal tolerance assays, plates were incubated at 30°C, 35°C, and 38°C, while osmotic stress assays were performed at 30°C with NaCl added to final concentrations of 0 M, 1 M, and 1.5 M. All experiments were performed in triplicate for each strain. All plates were incubated with continuous orbital shaking, and OD_600_ was recorded every 1 h for 24 h. Growth curves were obtained from OD measurements over time. Time values were converted to hours and growth data were log-transformed prior to analysis. The maximum specific growth rate (μmax) was estimated using a sliding-window linear regression approach over five consecutive time points, selecting the highest slope as μmax. The area under the curve (AUC) was calculated using the trapezoidal rule, and lag phase duration was estimated from the regression line corresponding to μmax. All calculations were performed independently for each replicate.

Growth parameters (maximum specific growth rate, μmax; lag phase; and area under the curve, AUC) were compared among strains within each environmental condition using one-way ANOVA. Homogeneity of variance was assessed using Levene’s test and normality of residuals using the Shapiro–Wilk test. Because each strain was represented by three biological replicates, the robustness of the statistical inference was further evaluated using Welch’s ANOVA and permutation-based ANOVA. Across all 18 condition × parameter combinations, the three statistical approaches produced concordant conclusions. Pairwise comparisons were performed using Tukey’s HSD post hoc test, and strains sharing the same letter were not considered significantly different (*P* < 0.05).

### Phenotypic microarray analysis of strain M6

Phenotypic characterization of carbon source utilization by the native *S. cerevisiae* strain M6 was performed using Biolog YT MicroPlates (Biolog Inc., USA), following the manufacturer’s instructions with minor modifications. Fresh yeast colonies grown on YPD agar were suspended in sterile saline solution. The cell suspension was adjusted to 47% transmittance prior to inoculation of the microplates.

Each well of the YT MicroPlate contains a different carbon source or metabolic substrate together with a tetrazolium-based redox dye. Utilization of a substrate results in cellular respiration and reduction of the dye, producing a purple color proportional to metabolic activity. Microplates were incubated at 28°C, and absorbance was measured at 590 nm using a Synergy H1X multi-mode plate reader (BioTek Instruments, USA). Corrected values were obtained by subtracting the corresponding control wells, and substrates with corrected responses above the defined threshold were considered positive.

## Results

### Yeast isolation and preliminary screening

**Figure 1:**
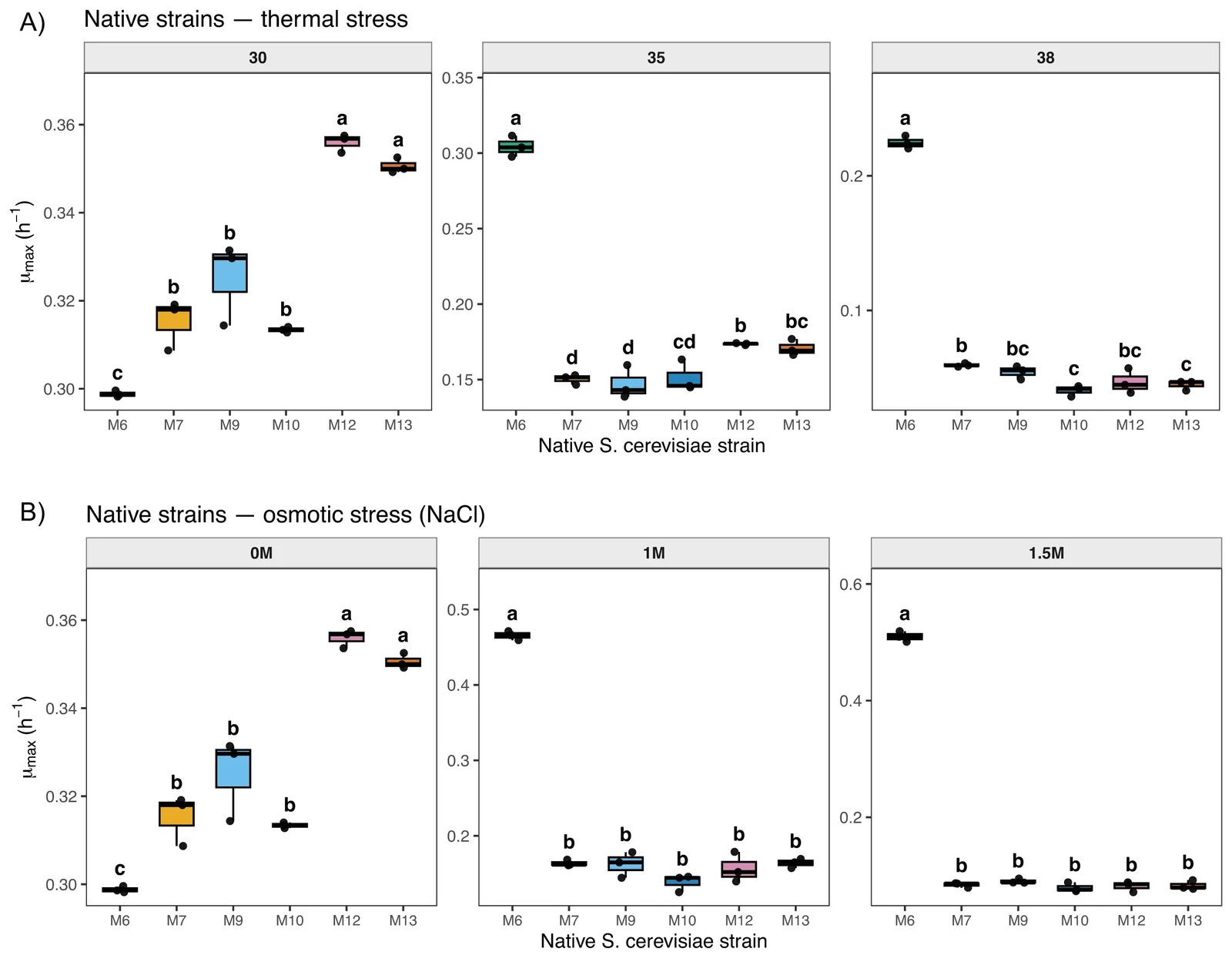
Maximum specific growth rate (μmax, h⁻¹) of native *S. cerevisiae* strains under osmotic and thermal stress conditions. Boxplots represent growth rates A) temperatures (30°C, 35°C, and 38°C) and B) at increasing NaCl concentrations (0 M, 1 M, and 1.5 M). Each boxplot shows the distribution of replicates for each strain. Different letters indicate significant differences among strains within each condition (*P* < 0.05). Statistical significance was assessed using one-way ANOVA followed by Tukey’s HSD post hoc test after verification of normality and homogeneity of variance assumptions.

A total of 10 yeast isolates were obtained from the grape must sample, including six *S. cerevisiae* strains, two *Metschnikowia spp*., one *Hanseniaspora sp*., and one *Pichia sp*. A preliminary screening was conducted to evaluate thermal and osmotic stress tolerance among the *S. cerevisiae* isolates, revealing that strain M6 exhibited the highest tolerance under both conditions. Among the evaluated strains, M6 consistently exhibited one of the highest μmax values under both thermal and osmotic stress conditions (Fig. 1). Under non-stress conditions (30°C), M6 was the slowest growing native isolate. In contrast, under thermal stress, M6 displayed the highest growth performance at both 35°C and 38°C. Under osmotic stress, M6 consistently exhibited the highest μmax values at 1 M and 1.5 M NaCl, while the growth of the remaining strains declined markedly with increasing salt concentration. These results indicate that the superior performance of M6 is stress-specific rather than a consequence of generally faster growth (Caspeta and Nielsen 2015), supporting its adaptation to both thermal and osmotic stress conditions (Fig. 1).

### Sequencing output and read preprocessing

Short-read sequencing of the M6 strain using the MGI platform generated a total of ∼3.14 million raw reads, which corresponded to an estimated raw coverage of ∼75×, assuming a genome size of 12 Mb. After quality filtering using a Q30 threshold, 1.91 million high-quality reads were retained for downstream analyses, confirming the overall high quality of the dataset.

For long-read sequencing, a total of 677 668 reads were obtained, yielding 4.70 Gb of raw data with an N50 read length of 8994 base pairs. Quality filtering indicated that the majority of reads had a quality score above Q10. Following additional filtering by read length and quality, 132 875 reads totaling 1.81 Gb were retained for genome assembly.

### Genome assembly and structural annotation

The final assembly consisted of 25 contigs with a total genome size of ∼11.89 Mb, providing an effective coverage of ∼48×. The largest contig reached 1.50 Mb in length, and the N50 value of the final assembly was 914 040 bp, indicating a high level of contiguity. Notably, the proportion of ambiguous bases (Ns) was reduced to zero after the final polishing step.

Completeness of the assembly was evaluated using BUSCO with the Saccharomycetes dataset. The final assembly achieved a BUSCO completeness score of 99.5%, indicating that nearly all expected single-copy orthologs were recovered. This high level of completeness supports the overall quality and reliability of the genome assembly for downstream annotation.

A total of 16 132 genes were initially predicted through ab initio methods. After refinement using Augustus with Saccharomycetes-specific parameters and additional manual curation, 5369 high-confidence genes were retained. This gene count is lower than those reported for recently published highly complete *S. cerevisiae* genomes, which have been estimated to contain an average of ∼6651 genes (Loegler et al. 2025). Differences in assembly structure, annotation pipelines, gene prediction parameters, and filtering criteria may contribute to variation in the final number of predicted genes. Nevertheless, the high completeness of the M6 assembly (BUSCO = 99.5%) indicates that the vast majority of conserved single-copy orthologs are represented in the genome.

### Phylogenetic analysis and genomic characterization of the M6 strain

Phylogenetic analysis based on ITS sequences confirmed the taxonomic identity of the M6 strain as *S. cerevisiae*. M6 clustered tightly with other *S. cerevisiae* strains, forming a well-supported monophyletic group with high bootstrap values (≥0.83). The tree topology clearly separates *S. cerevisiae* from closely related species such as S. paradoxus and S. kudriavzevii. It is consistent with previous ITS-based phylogenies of the genus, further supporting the assignment of M6 to *S. cerevisiae* (Supplementary Fig. 1).

To complement the phylogenetic analysis, an Average Nucleotide Identity (ANI) comparison between the M6 genome and the reference strain *S. cerevisiae* S288C revealed a 99.42% nucleotide identity, further confirming its taxonomic assignment within *S. cerevisiae*.

### Ploidy estimation

Flow cytometry analysis using propidium iodide staining indicated that strain M6 exhibits a DNA content consistent with a diploid genome. The fluorescence peak of M6 was located close to, but slightly shifted relative to, the diploid control, and clearly distinct from the tetraploid control (Supplementary Fig. 2).

Allele balance distribution of heterozygous SNPs showed a strong peak centered around 0.5, consistent with diploid ploidy. Furthermore, the normalized genome-wide coverage profile showed relatively uniform sequencing depth across the genome with values close to the expected diploid baseline, and no chromosome-scale deviations indicative of aneuploidy were observed (Supplementary Fig. 3).

Additionally, ploidy estimation based on variant calling identified 6821 total alleles, of which 6615 (97%) were heterozygous and 206 homozygous. This high proportion of heterozygous sites strongly supports a diploid genome configuration for the native *S. cerevisiae* M6 strain. These observations support a predominantly diploid genetic background.

### Local divergence of the M6 strain within the Wine/European lineage

**Figure 2:**
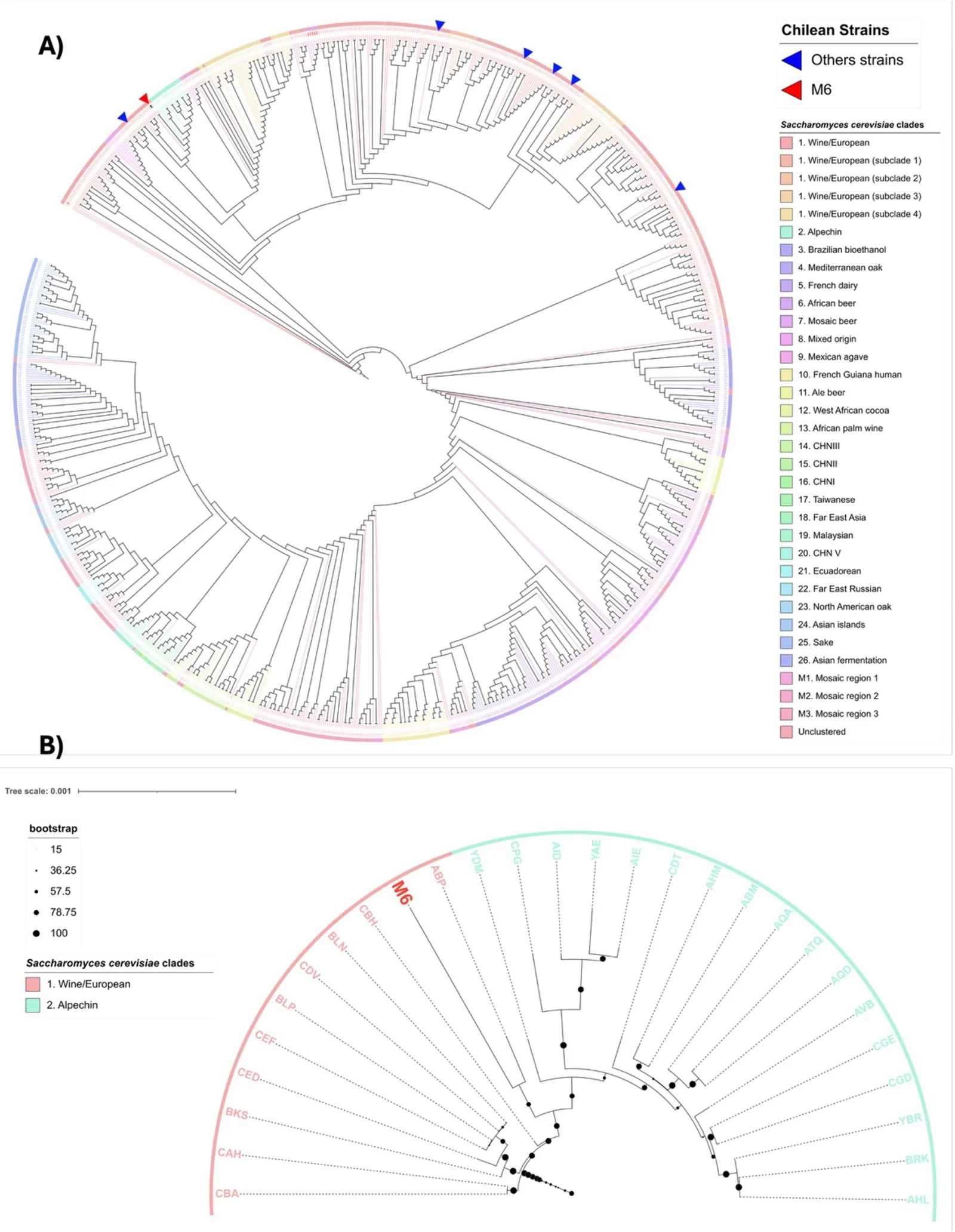
Maximum-likelihood phylogeny of *S. cerevisiae* M6 based on 67 concatenated CDS from the 1011 genomes reported by Peter et al. (2018). The tree was inferred with IQ-TREE (GTR + R, 1000 ultrafast bootstraps). (A) Circular tree showing Chilean strains and M6 within global clades, as indicated by the labeled arrowheads (see inset legend).(B) Subtree highlighting M6 clustering in the Wine/European clade near Alpechin strains.

The M6 strain falls within the Wine/European lineage (lineage 1, Fig. 2), sharing a clear evolutionary origin with other domesticated strains, as evidenced by its close phylogenetic clustering with strain ABP—isolated from prickly pear (Opuntia) fruit in Spain—with a bootstrap support of 73% and a genetic distance of 0.16 (Supplementary Table 2). This proximity underscores M6’s phylogenetic stability within a widely distributed domesticated group, while its higher average divergence (0.53) compared to ABP (0.43) and to the mean observed among six Chilean strains (0.41). M6’s divergence remains well below that of highly distinct lineages, such as the Taiwanese (>1.80). Such divergence may be associated with the environmental conditions of its origin—marked by high salinity, elevated temperatures, and nutrient scarcity—although further functional and comparative analyses would be required to determine any adaptive significance. Importantly, M6’s divergence remains well below that of highly distinct lineages, supporting its placement within a globally recognized lineage (Fig. 2; Supplementary Fig. 4).

### Sequence variants in genes associated with environmental stress

A total of 56 977 genomic variants were identified in the M6 strain when compared to the reference genome of *S. cerevisiae* S288C. Of these, 47 157 (82.8%) were classified as single nucleotide polymorphisms (SNPs), while 9820 (17.2%) corresponded to insertions or deletions (INDELs). Functional consequences were inferred from predicted effects on coding regions and gene structure. The most common variant types were synonymous mutations (28.75%) and missense mutations (15.96%), followed by variants located in intronic regions (1.1%).

To further evaluate the genomic distinctiveness of M6, a comparative SNP analysis was performed using publicly available wine-associated (EC1118 and DBVPG3051) and Alpechin (CBS2909 and YJM1078) *S. cerevisiae* strains (Supplementary Table 3). A total of 136 969 polymorphic sites were identified across the five strains. SNP-based phylogenetic clustering separated the Alpechin strains from the wine-associated lineage and placed M6 within the wine-associated group, where it clustered most closely with DBVPG3051. These results support a closer genomic relationship of M6 to wine-associated strains than to the Alpechin lineage.(Supplementary Fig. 5).

Joint variant calling identified 4904 variants unique to M6, including 4800 SNPs and 104 INDELs. Functional annotation revealed 1655 missense variants, 1335 synonymous variants, and 18 predicted high-impact variants, including frameshift, stop-gained, stop-lost, and start-lost mutations.

Among genes associated with environmental stress adaptation, several M6-specific variants were retained following comparative analysis. These included missense substitutions in components of the HOG signaling pathway (*SSK1*, p.Pro461Ala; *SSK2*, p.Asp1004Ala), trehalose metabolism (*TPS2*, p.Ser764Arg; *NTH1*, p.Asn559Asp), heat-shock and proteostasis-related genes (*HSP104*, p.Val641Ile; *HSC82*, p.Val704Ile), and membrane-associated genes (*ERG7*, p.Ile352Met; *ERG20*, p.Val15Ile and p.Leu322Met). Additional high-impact variants were identified in regulatory genes including *GAL4* (stop gained), *PSK2* (stop lost), *KIN4* (stop gained), *FRE4* (stop gained), and *MET4* (frameshift) (Table 1).

**Table 1:** Functionally annotated variants unique to strain M6 identified through comparative genomic analysis with wine-associated and Alpechin *S. cerevisiae* strains.

Functional category	Gene	Variant type	Variant(s) unique to M6
HOG signaling	*SSK1*	Missense	p.Pro461Ala
HOG signaling	*SSK2*	Missense	p.Asp1004Ala
Trehalose metabolism	*TPS2*	Missense	p.Ser764Arg
Trehalose degradation	*NTH1*	Missense	p.Asn559Asp
Heat shock response	*HSP104*	Missense	p.Val641Ile
Molecular chaperone	*HSC82*	Missense	p.Val704Ile
Ergosterol biosynthesis	*ERG7*	Missense	p.Ile352Met
Isoprenoid biosynthesis	*ERG20*	Missense	p.Val15Ile; p.Leu322Met
Sulfur metabolism	*MET4*	Frameshift	p.Gln605fs
Carbon utilization/regulation	*GAL4*	Stop gained	p.Gln752*
Cell cycle regulation	*KIN4*	Stop gained	p.Gln158*
Iron homeostasis	*FRE4*	Stop gained	p.Arg514*
Nutrient signaling	*PSK2*	Stop lost	p.*1102Trp

Although these variants represent candidate determinants of the distinctive phenotype of strain M6, their functional consequences remain putative and require experimental validation.

To evaluate potential genomic signatures associated with adaptation to anthropic fermentation environments, we examined the SSU1 and CUP1 loci. A homolog of SSU1, associated with sulfite resistance, was identified in the M6 genome assembly with 99.6% identity over 1377 bp. Analysis of the genomic region surrounding SSU1 revealed a conserved syntenic organization, with NOG1 (YPL093W) immediately upstream and GLR1 (YPL091W) immediately downstream, matching the canonical arrangement described for chromosome XVI in the S288C reference genome (Supplementary Table 4). No evidence of major structural rearrangements affecting the immediate SSU1 locus was detected. In addition, BLAST searches revealed at least six tandem copies of the CUP1 gene within the same contig, consistent with the copy number expansion of this copper tolerance locus described in many wine-associated *S. cerevisiae* strains.

### Genome assembly structure and identification of unassigned regions

After *de novo* assembly and reference-guided scaffolding using RagTag with the *S. cerevisiae* S288C genome, a total of 24 contigs were recovered. Of these, 16 were confidently anchored to the 16 canonical nuclear chromosomes of *S. cerevisiae*, confirming the accuracy and completeness of the assembly (Supplementary Table 5). The remaining six contigs were analyzed using BLAST alignments to determine their origin. Three contigs were classified as plasmid-derived, including one corresponding to the complete 2 μm plasmid (contig_29_pilon) and two partial fragments with high similarity to the *S. cerevisiae* 2 μm plasmid (contigs_30_pilon and _38_pilon). Two contigs were identified as mitochondrial in origin—one complete (contig_2_pilon) and one fragmentary (contig_3_pilon).

The remaining contigs, contig_36_pilon and contig_39_pilon, did not align to the S288C nuclear genome but showed partial similarity to regions in chromosomes I, VI, XI, and XIV in other *S. cerevisiae* strains. Notably, the genes identified in contig_39_pilon corresponded to telomeric sequences, characterized by high levels of repetitive motifs, suggesting their association with chromosome ends. These contigs likely represent structurally variable or strain-specific regions absent or underrepresented in the reference genome, and were therefore retained for further analysis.

Gene prediction analysis of contig_36_pilon identified seven coding sequences (CDSs) (Supplementary Table 6). Comparative analysis against the *S. cerevisiae* pangenome showed that all predicted genes exhibited high sequence identity and coverage to previously described loci, indicating that they correspond to conserved genes located in non-aligned genomic regions rather than novel M6-specific genes. Several of the genes identified in contig_36_pilon are associated with metabolic and stress-response functions previously described in *S. cerevisiae*. These include genes related to thiamine biosynthesis (*THI5*), aldehyde and alcohol metabolism (*AAD6* and *DAK2*), amino acid and nitrogen utilization (*AGP3*), osmotic regulation through aquaglyceroporin activity (*AQY3*), and transcriptional regulation associated with carbon metabolism and stress adaptation (*ZNF1*). The presence of these genes within non-aligned or subtelomeric regions is consistent with the dynamic nature of subtelomeric domains in yeast genomes, which are often enriched in environmentally responsive genes and exhibit high structural variability among strains.

### Growth performance of M6 under thermal and osmotic stress

**Figure 3:**
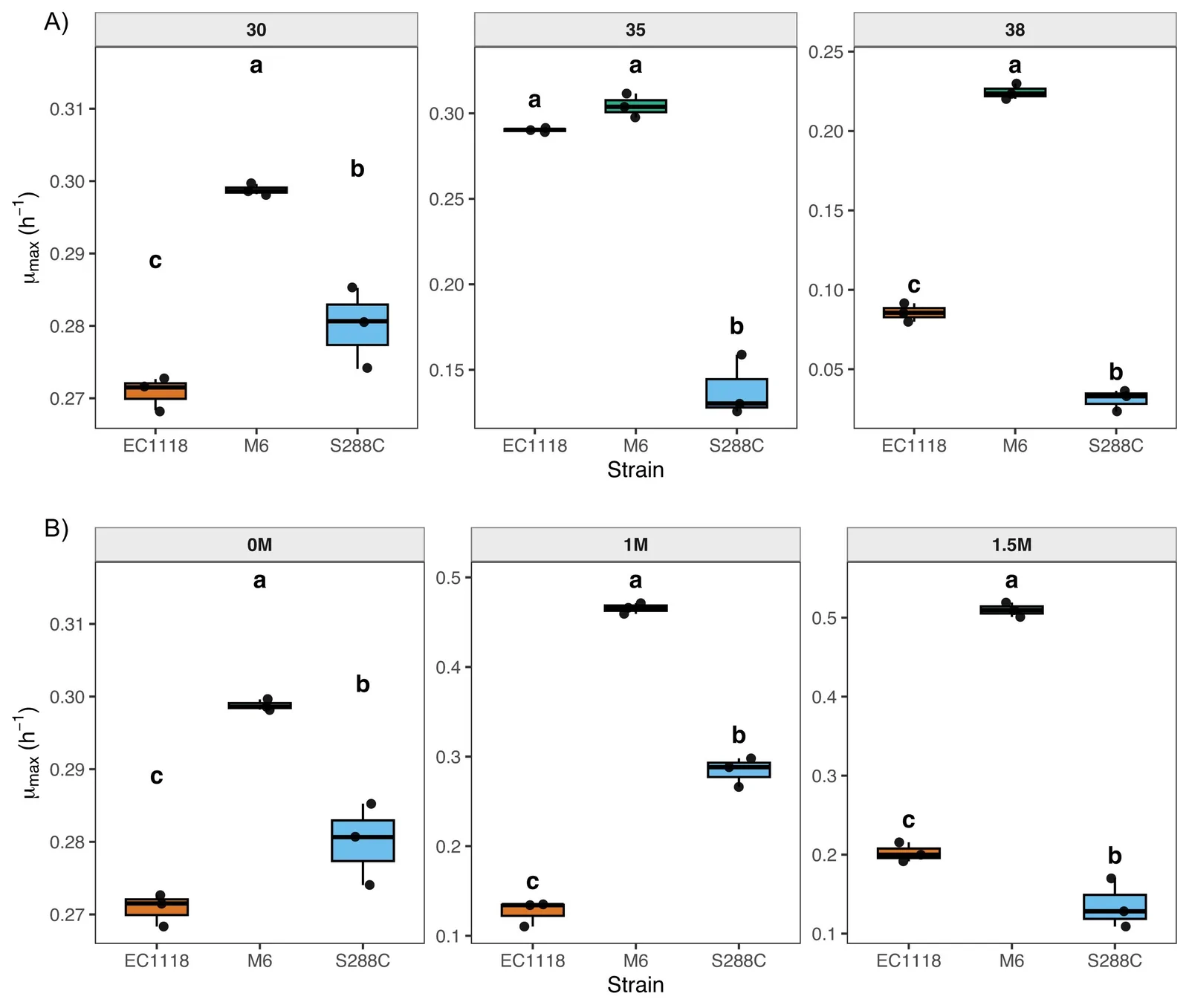
Maximum specific growth rate (μmax) of *S. cerevisiae* strains under thermal and osmotic stress conditions. Growth rate of three yeast strains (*S. cerevisiae* M6, EC1118, and S288C) at 30°C, 35°C, and 38°C (upper panel) and at 0 M, 1 M, and 1.5 M of NaCl (bottom panel). Boxplots show the distribution of replicate measurements for each condition. Different letters indicate significant differences among strains within each condition (*P* < 0.05), based on one-way ANOVA followed by Tukey’s HSD post hoc test.

The genomic signals of adaptation identified in M6 were supported by phenotypic assays evaluating the maximum specific growth rate (μmax) under abiotic stress conditions. At 30°C, M6 already displayed a significantly higher μmax than both EC1118 and S288C. At 38°C, M6 maintained significantly higher μmax values than EC1118 and S288C, whereas at 35°C M6 did not differ significantly from EC1118. At 38°C, M6 exhibited ∼2.5-fold higher μmax than EC1118 and more than five-fold higher μmax than S288C (Fig. 3; upper panel).

Similarly, under osmotic stress, M6 showed the highest tolerance to NaCl. Even in the absence of salt, M6 grew significantly faster than EC1118 and S288C, and it maintained substantially higher growth rates at both 1 M and 1.5 M NaCl. At 1.5 M NaCl, μmax values in M6 were ∼2.5-fold higher than in EC1118 and nearly four-fold higher than in S288C (Fig. 3; bottom panel). In contrast, growth of both reference strains declined markedly with increasing salinity, with S288C showing the strongest inhibition. These results further support the enhanced osmotic stress tolerance of M6.

Analysis of growth dynamics revealed significant differences among the evaluated *S. cerevisiae* strains under both osmotic and thermal stress conditions (Supplementary Table 7). Strain M6 generally exhibited higher overall growth performance, reflected by increased μmax and AUC values under elevated salinity and temperature conditions, particularly at 1–1.5 M NaCl and 38°C. Differences in lag phase were also observed under several stress conditions; however, these effects were less consistent than those observed for μmax and AUC (Supplementary Fig. 6 and 7).

### Phenotypic characterization of carbohydrate utilization in strain M6

**Figure 4:**
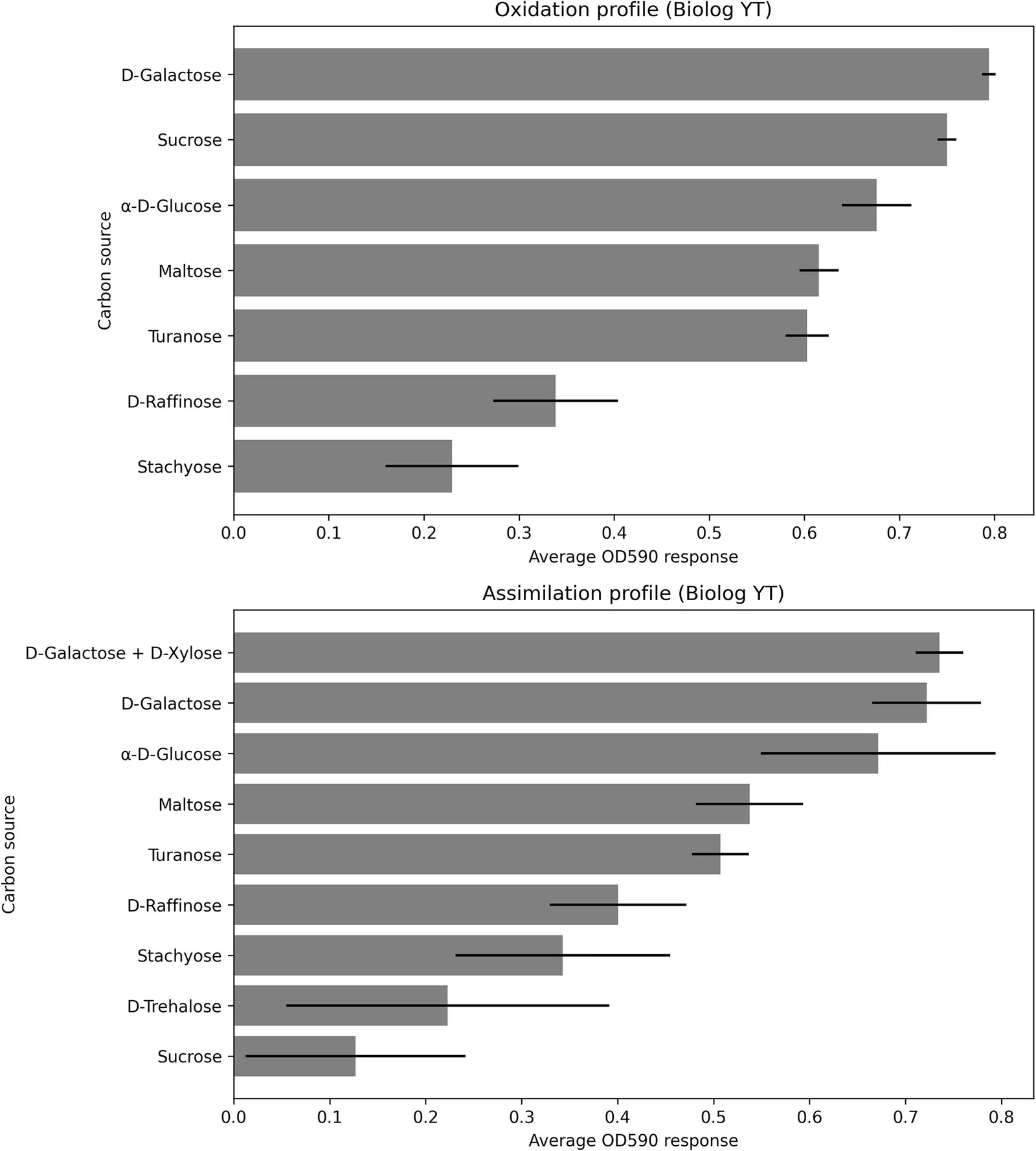
Oxidation and assimilation profiles of carbon substrates in *S. cerevisiae* strain M6 determined using Biolog YT MicroPlates™. Bar plots represent the average OD590 response values obtained from duplicate assays, while error bars indicate standard deviation. The upper panel corresponds to oxidative metabolism assays and the lower panel to substrate assimilation assays. Only substrates showing positive responses above the selected threshold are displayed.

Biolog YT assays revealed that strain M6 exhibited a broad capacity to oxidize and assimilate diverse carbohydrate substrates, with particularly strong responses toward simple sugars and selected oligosaccharides (Fig. 4). In the oxidation profile, the highest responses were observed for D-galactose, sucrose, α-D-glucose, maltose, and turanose, indicating an active oxidative metabolism associated with mono- and disaccharide utilization. Moderate oxidative responses were also detected for D-raffinose and stachyose, suggesting the ability of M6 to partially metabolize raffinose-family oligosaccharides. Similarly, assimilation assays showed strong utilization of D-galactose, α-D-glucose, maltose, and D-galactose + D-xylose, while moderate assimilation was observed for D-raffinose, stachyose, and D-trehalose. Overall, these results indicate that M6 possesses a versatile carbohydrate utilization profile, including the capacity to metabolize complex plant-associated sugars and oligosaccharides potentially relevant under fermentative and stress-associated environmental conditions.

## Discussion

The genomic characterization of the native *S. cerevisiae* strain M6, isolated from grape must from a vineyard located in the Atacama Desert, provides novel insights into the mechanisms by which yeast adapts to extreme environments. The high-quality *de novo* hybrid assembly, with 99.5% completeness and strong contiguity (N50: 914 kb), supports the robustness of downstream analyses and enables a detailed exploration of genetic variations potentially linked to performance under high salinity, elevated temperatures, and nutrient-limited conditions.

### Genomic divergence within the Wine/European lineage

Phylogenomic analysis placed M6 within the Wine/European lineage, in close relationship with strain ABP, isolated from prickly pear (Opuntia) fruit in Spain, but with greater genetic distance than the average observed among Chilean strains analyzed (Supplementary Table 8). This combination of phylogenetic proximity and genomic divergence suggests that M6 shares a common domesticated origin while exhibiting distinctive genomic and phenotypic features in the context of the extreme environmental conditions Atacama Desert. Interestingly, the closest related strains identified in the phylogenetic reconstruction include isolates such as CBH and BLN, which were originally recovered from grape must and wine-associated environments in Israel and Spain, respectively. This clustering suggests that despite the extreme environmental conditions of the Atacama Desert, M6 retains genomic signatures characteristic of wine-associated *S. cerevisiae* populations. In contrast, strains belonging to the Alpechin clade are primarily linked to olive-related fermentative environments and other industrial niches, highlighting the ecological differentiation within domesticated *S. cerevisiae* lineages.

Local genetic structuring have been reported in other contexts of microbial terroir. For instance, studies from in New Zealand have shown that *S. cerevisiae* populations can exhibit region-specific population structure despite ongoing gene flow (Gayevskiy et al. 2012), and genetically distinct or mixed yeast communities can differentially shape wine aroma and regional sensory attributes (Swieboda et al. 2015, Hawkins et al. 2023).

### Variants in stress-response genes

**Figure 5:**
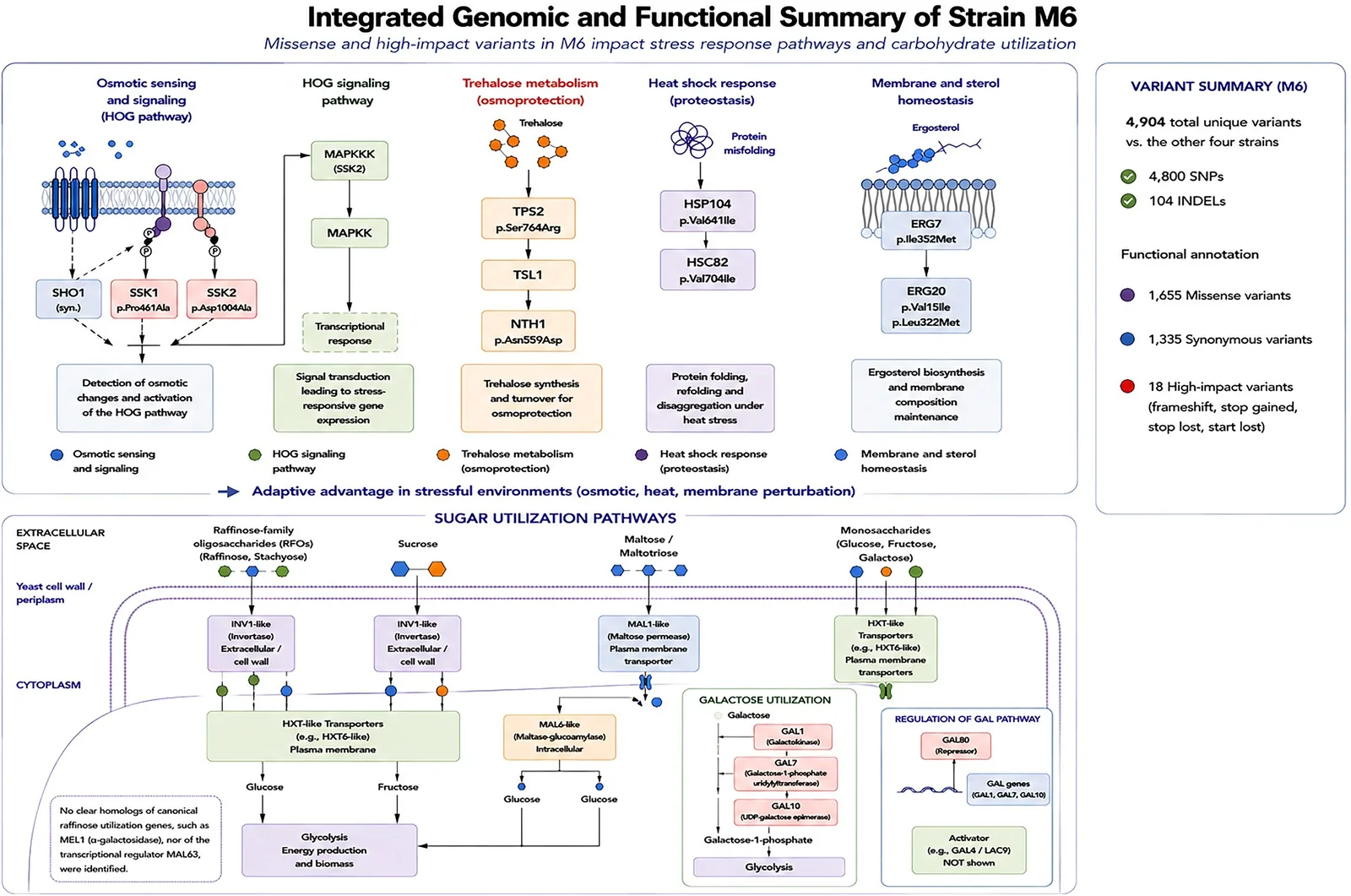
Integrated genomic and functional summary of strain M6. Schematic representation of candidate pathways potentially associated with the distinctive phenotype of *S. cerevisiae* strain M6 based on comparative genomic analysis and phenotypic characterization. Unique missense variants identified in M6 are highlighted in genes involved in osmotic sensing and HOG signaling (SSK1, SSK2), trehalose metabolism (TPS2, NTH1), heat-shock response and proteostasis (HSP104, HSC82), and membrane sterol homeostasis (ERG7, ERG20). The lower panel summarizes putative carbohydrate utilization pathways inferred from genomic annotation, including transport and metabolism of mono-, di-, and oligosaccharides. Created using AI-assisted graphical tools and manually curated by the authors.

Comparative analysis of stress-response loci identified coding variants in genes involved in osmotic sensing and signaling (*SSK1, SSK2*), trehalose metabolism (*TPS2, NTH1*), and proteostasis and heat-shock response (*HSP104, HSC82*). No premature stop codons or other obvious loss-of-function mutations were detected in these genes (Fig. 5). These findings are consistent with previous studies showing that stress adaptation in *S. cerevisiae* frequently involves the accumulation of mutations across interconnected regulatory and stress-response pathways, including the HOG signaling pathway, cell wall integrity systems, and proteostasis networks, rather than adaptation driven by single gene-specific changes (Voordeckers et al. 2015, Huang et al. 2018, Gan et al. 2022). Collectively, these observations suggest that strain M6 harbors genomic variations that may contribute to its performance under the abiotic conditions tested.

### Unassigned genomic elements and adaptive potential

The comparative analysis of contig_36 using AUGUSTUS gene prediction revealed the presence of seven predicted coding sequences, all corresponding to conserved loci previously described in the *S. cerevisiae* pangenome. Several of these genes are associated with stress adaptation and metabolic regulation. For instance, *AQY3* encodes an aquaglyceroporin involved in glycerol transport and osmotic balance, while *DAK2* participates in glycerol metabolism through dihydroxyacetone kinase activity. Likewise, *ZNF1* is a transcriptional regulator associated with carbon metabolism and adaptation to nutrient limitation, and *AGP3* encodes a plasma membrane transporter potentially involved in amino acid uptake. Together, these findings suggest that contig_36 contains conserved stress- and metabolism-related loci that may contribute to the physiological flexibility of the M6 strain under harsh fermentation-associated conditions such as osmotic stress, nutrient limitation, and temperature fluctuations (Boer et al. 2010).

### Phenotypic validation of stress tolerance

The genomic signals of adaptation identified in M6 were supported by phenotypic assays that directly evaluated the maximum specific growth rate (μmax) under abiotic stress. M6 already grew significantly faster than EC1118 and S288C at 30°C, and this advantage increased under thermal stress. At 38°C, M6 maintained significantly higher μmax values than EC1118 and S288C, whereas at 35°C it did not differ significantly from EC1118, indicating enhanced thermotolerance that becomes evident at the highest temperature. At 38°C, M6 exhibited μmax values ∼2.5-fold higher than EC1118 and more than five-fold higher than S288C.

Similarly, M6 grew faster than EC1118 and S288C even in the absence of salt, and it maintained substantially higher growth rates at both 1 M and 1.5 M NaCl. At 1.5 M NaCl, M6 exhibited μmax values ∼2.5-fold higher than EC1118 and nearly four-fold higher than S288C. These findings demonstrate that the distinctive genomic features identified in M6 are accompanied by enhanced tolerance to osmotic and thermal stress. Importantly, this evidence reinforces its potential as a resilient starter culture under climate-stressed winemaking scenarios.

### Integrated phenotypic and genomic analysis of carbon metabolism in M6

Phenotypic assays indicated that strain M6 is capable of utilizing raffinose, stachyose, and turanose, suggesting a degree of flexibility in carbon source utilization. Genomic analysis identified an invertase-encoding gene (*INV1*-like; EC 3.2.1.26), together with a functional maltose utilization system comprising a sugar transporter (*MAL1*-like) and an α-glucosidase (*MAL6*-like; *GH13*/*GH31*), as well as multiple transporters belonging to the major facilitator superfamily (e.g. *HXT5*-like proteins), supporting the uptake and metabolism of mono- and disaccharides (Fig. 5). However, no clear homologs of canonical raffinose utilization genes, such as *MEL1* (α-galactosidase), nor of the transcriptional regulator *MAL63*, were identified. In *S. cerevisiae*, raffinose is first hydrolyzed by extracellular invertase (*SUC2*/*INV1*), releasing fructose and melibiose. Complete utilization of raffinose requires *MEL1*-mediated hydrolysis of melibiose into glucose and galactose. The absence of a detectable *MEL1* homolog in M6 suggests that raffinose metabolism may be incomplete, with growth supported primarily by the uptake and utilization of fructose released during the initial hydrolysis step. These results are consistent with a model in which M6 relies on partial hydrolysis of raffinose-family oligosaccharides and subsequent utilization of released monosaccharides rather than complete degradation of these substrates. In contrast to strains such as *MT1*, an *S. cerevisiae* strain isolated from the fermentation process used for the production of Chinese Maotai-flavored liquor, which harbors *MEL1* and exhibits coordinated multi-carbon utilization (Lu et al. 2015), M6 appears to employ a less specialized but functionally effective metabolic strategy. The presence of an *RTG1*-like transcriptional regulator further suggests a contribution of global metabolic regulation to the observed phenotype. The possible involvement of uncharacterized glycoside hydrolases in the utilization of these substrates cannot be excluded.

The presence of *GAL1, GAL10*, and *GAL80* homologs indicates that strain M6 harbors a functional galactose utilization pathway, consistent with its ability to grow on D-galactose. Although a heterozygous stop-gained variant (Q752) was identified in one allele of the transcriptional regulator GAL4, the second allele remains intact, which is consistent with the observed galactose utilization phenotype. This suggests that, in addition to glucose and fructose, M6 may also metabolize galactose released from raffinose-family oligosaccharides, further contributing to its metabolic flexibility.

### Biotechnological implications and outlook

The suite of adaptations detected in M6, combined with its membership in a globally recognized wine lineage, positions M6 as a valuable biological resource for investigating the genetic and regulatory bases of yeast performance under combined thermal and osmotic stress. Beyond its genomic signatures, growth assays demonstrated that M6 sustains significantly higher growth rates than commercial (EC1118) and laboratory (S288C) strains under elevated temperatures and high osmotic stress, validating its superior stress tolerance at the phenotypic level. This dual evidence highlights the relevance of M6 as a model system to explore how naturally occurring genetic variation contributes to stress tolerance in wine-associated yeast lineages, with potential implications for understanding yeast adaptation in climate-stressed environments

## Supplementary Material

foag033_Supplemental_Files

## Data Availability

Raw sequencing reads are available under accessions ERR15464771 (short reads) and ERR17722420 (Oxford Nanopore long reads). The genome assembly of *S. cerevisiae* strain M6 has been deposited in the European Nucleotide Archive (ENA) under BioProject PRJEB96299, BioSample SAMEA119586790, and assembly accession GCA_966887395.
