# Supplementary material for: De novo genome assembly of a native Saccharomyces cerevisiae strain isolated from spontaneous fermentation of vineyard must in the Atacama Desert (Chile)
Source: FEMS Yeast Res. 2026 Aug 4;26:foag033. doi: 10.1093/femsyr/foag033 (PMC13495792; doi:10.1093/femsyr/foag033)
Supplement: Supplementary file 1 [file foag033_supplemental_files.zip › Supplementary_figures_v4.docx]

**Supplementary Figure 1.** Maximum likelihood phylogenetic tree based on ITS sequences showing the placement of the M6 strain within the Saccharomyces cerevisiae clade. Bootstrap support values are represented by proportional black circles at each node (see legend).

Diploid

Tetraploid

M6

**Supplementary Figure 2.** DNA content profiles for ploidy estimation. Flow cytometry histograms for diploid and tetraploid controls and strain M6. The M6 profile is consistent with diploidy.

**Supplementary Figure 3**. Genomic inference of ploidy in strain M6. (A) Distribution of allele balance for heterozygous SNPs (DP ≥ 20). Allele balance represents the fraction of sequencing reads supporting the alternative allele at heterozygous variant positions. Only SNPs with sequencing depth ≥20 were considered. (B) Genome-wide normalized sequencing coverage of strain M6 mapped to the *Saccharomyces cerevisiae* EC1118 reference genome. Coverage was calculated from read alignments and summarized in 10 kb genomic windows.

**Supplementary Figure 4.** Circular phylogenetic tree showing the population structure and lineage assignment of Saccharomyces cerevisiae strains included in the comparative genomic analysis. Branches are colored according to the clade classification described by Peter et al. (2018), including Wine/European, Asian, African, beer-associated, and mosaic lineages.

**Figure 5. SNP-based Neighbor-Joining tree of strain M6 and four publicly available Saccharomyces cerevisiae strains.** The tree was constructed from 136,969 polymorphic sites identified by joint variant calling against the S288C reference genome. M6 clustered within the wine-associated lineage and showed the closest relationship to DBVPG3051, whereas the Alpechin strains (CBS2909 and YJM1078) formed a distinct cluster.

A)

B)

**Supplementary Figure 6.** Supplementary Figure 7. Comparative growth performance of *S. cerevisiae* strains under osmotic and thermal stress conditions. (A) Area under the growth curve (AUC; OD × h) of strains M6, EC1118, and S288C under increasing NaCl concentrations (0, 1, and 1.5 M). (B) AUC values under increasing temperature stress (30°C, 35°C, and 38°C). Boxplots represent biological replicates. Statistical analyses, including ANOVA, Welch’s ANOVA, and post hoc comparisons, are provided in Supplementary Table 6.

A)

B)

**Supplementary Figure 7.** Comparative lag phase duration of *S. cerevisiae* strains under osmotic and thermal stress conditions. (A) Lag phase duration (h) of strains M6, EC1118, and S288C under increasing NaCl concentrations (0, 1, and 1.5 M). (B) Lag phase duration (h) under increasing temperature stress (30°C, 35°C, and 38°C). Boxplots represent biological replicates. Statistical analyses, including ANOVA, Welch’s ANOVA, and post hoc comparisons, are provided in Supplementary Table 6.

**Supplementary Figure 8.** Growth curves of *Saccharomyces* cerevisiae strain M6 and reference strains S288C and EC1118 under osmotic and thermal stress conditions. Growth was monitored by OD₆₀₀ measurements over 24 h in YPD medium supplemented with different NaCl concentrations (0 M, 1 M, and 1.5 M) and at different incubation temperatures (30 °C, 35 °C, and 38 °C).
