# Supplementary material for: De novo genome assembly of a native Saccharomyces cerevisiae strain isolated from spontaneous fermentation of vineyard must in the Atacama Desert (Chile)
Source: FEMS Yeast Res. 2026 Aug 4;26:foag033. doi: 10.1093/femsyr/foag033 (PMC13495792; doi:10.1093/femsyr/foag033)
Supplement: Supplementary file 1 [file foag033_supplemental_files.zip › Supplementary_Table_v4.docx]

**Supplementary Table 1. List of 67 Genes Used for Phylogenetic Reconstruction.**

**This table provides the identifiers of the selected genes, their average nucleotide length, the proportion of strains with full-length representation for each gene, and the percentage of phylogenetically informative sites. These genes correspond to coding sequences (CDS) extracted from the dataset of 1,011 *Saccharomyces cerevisiae* genomes published by Peter et al. (2018). Gene selection was based on the presence of single-copy, highly conserved genes across all strains, aiming to minimize the impact of gene duplication or loss events and to** **maximize the phylogenetic signal. Each gene was individually aligned using MAFFT v7.525 and subsequently concatenated into a supermatrix using AMAS for phylogenetic tree construction.**

**Supplementary Table 2.** Strains included in the phylogenetic analysis (Figure 2B) and their ecological and geographical origins. Standardized names follow the nomenclature of Peter et al. (2018). The dataset includes strains from the Wine/European and Alpechin clades, representing diverse ecological niches such as grape must, soil, olive-related environments, and human-associated sources.

| **Standardized name** | **Isolate name** | **Ecological origin** | **Substrate / Source** | **Geographical origin** | **Clade** |
| --- | --- | --- | --- | --- | --- |
| CHB | Lib54 | Wine | Fermenting grape must (white wine) | Lebanon | Wine/European |
| ABP | CECT10109_1b | Fruit | Prickly pear | Spain | Wine/European |
| CBH | DBVPG3051 | Wine | Grape must | Israel | Wine/European |
| BLN | DBVPG1659 | Wine | Grape must | Spain (La Mancha) | Wine/European |
| CDV | DBVPG1616-1D | Soil | Soil | Spain | Wine/European |
| BLP | DBVPG1661 | Wine | Grape must | Spain (La Mancha) | Wine/European |
| CEF | DBVPG1623-2A | Soil | Soil | Spain | Wine/European |
| CED | DBVPG1621-5A | Soil | Soil | Spain | Wine/European |
| BKS | DBVPG1595 | Wine | Grape must | Spain (La Mancha) | Wine/European |
| CAH | D-1 | Wine | Wine (amphora) | Georgia | Wine/European |
| CBA | A-14 | Wine | Wine (amphora) | Georgia | Wine/European |
| SACE_YDM | YJM1078 | Human clinical | Clinical isolate | Portugal | Alpechin |
| CPG | 1560 | Natural | Olive (Manzanilla-Alorena) | Spain | Alpechin |
| AID | CBS2909 | Human | Feces | NA | Alpechin |
| SACE_YAE | YJM248 | Human | Feces | NA | Alpechin |
| AIE | CBS2910 | Human | Feces | Portugal | Alpechin |
| CDT | CECT10171-5B | Insect | *Dacus oleae* larvae | Spain | Alpechin |
| AHM | CBS3093 | Industrial | Alpechin (olive wastewater) | Spain | Alpechin |
| AQA | CBS4903 | Industrial | Alpechin (olive wastewater) | Spain | Alpechin |
| ATQ | CECT10497 | Industrial | Alpechin (olive wastewater) | Spain | Alpechin |
| AQD | CBS5378 | Industrial | Alpechin (olive wastewater) | Spain | Alpechin |
| AVB | CECT10266 | Industrial | Tanning liquor | Spain | Alpechin |
| CGE | UCD_11-601 | Fruit | Olive fermentation | USA (Davis, CA) | Alpechin |
| CGD | UCD_09-448 | Fruit | Olives | USA (Davis, CA) | Alpechin |
| SACE_YBR | YJM1252 | Industrial | Alpechin (olive wastewater) | Spain | Alpechin |
| BRK | DBVPG6302 | Industrial | Alpechin (olive wastewater) | Spain | Alpechin |
| AHL | CBS3081 | Industrial | Alpechin (olive wastewater) | Spain | Alpechin |

**Supplementary Table 3.** Publicly available *Saccharomyces cerevisiae* strains used for comparative genomic analyses. Short-read sequencing datasets corresponding to wine-associated and Alpechin lineages were retrieved from public repositories and used for joint variant calling and phylogenetic analyses.

| **Strain** | **Accession** | **Reads (#)** | **Bases (Gb)** | **Lineage** |
| --- | --- | --- | --- | --- |
| YJM1078 | SRR800854 | 4,676,349 | 0.94 | Alpechin |
| DBVPG3051 | ERR1308628 | 7,268,185 | 1.40 | Wine/European |
| CBS2909 | ERR1309135 | 15,718,615 | 3.10 | Alpechin |
| EC1118 | ERR560555 | 24,597,930 | 4.90 | Wine/European |

**Supplementary Table 4.** Conservation of the genomic neighborhood surrounding SSU1 in M6 and the S288C reference genome. The genes flanking SSU1 in M6 correspond to the orthologous NOG1 (YPL093W) and GLR1 (YPL091W) loci in S288C, preserving the canonical NOG1–SSU1–GLR1 arrangement.

**M6 gene ID	M6 coordinates	Ortholog (S288C)	S288C coordinates**

g286.t1	367,696–369,639	NOG1 (YPL093W)	370,978–372,921

g287.t1	370,511–371,887	SSU1 (YPL092W)	373,793–375,169

g288.t1	372,268–373,671	GLR1 (YPL091W)	375,502–376,953

**Supplementary Table 5**. Classification and Interpretation of Contigs in the *Saccharomyces cerevisiae* Assembly After Reordering with RagTag

| Contig | Classification | Location / Alignment | No. of Genes | Interpretation |
| --- | --- | --- | --- | --- |
| BK006934.2_RagTag | Nuclear chromosome | Chromosome I (S288C) | 540 | Correctly anchored |
| BK006935.2_RagTag | Nuclear chromosome | Chromosome II (S288C) | 410 | Correctly anchored |
| BK006936.2_RagTag | Nuclear chromosome | Chromosome III (S288C) | 290 | Correctly anchored |
| BK006937.2_RagTag | Nuclear chromosome | Chromosome IV (S288C) | 580 | Correctly anchored |
| BK006938.2_RagTag | Nuclear chromosome | Chromosome V (S288C) | 360 | Correctly anchored |
| BK006939.2_RagTag | Nuclear chromosome | Chromosome VI (S288C) | 210 | Correctly anchored |
| BK006940.2_RagTag | Nuclear chromosome | Chromosome VII (S288C) | 340 | Correctly anchored |
| BK006941.2_RagTag | Nuclear chromosome | Chromosome VIII (S288C) | 250 | Correctly anchored |
| BK006942.2_RagTag | Nuclear chromosome | Chromosome IX (S288C) | 280 | Correctly anchored |
| BK006943.2_RagTag | Nuclear chromosome | Chromosome X (S288C) | 300 | Correctly anchored |
| BK006944.2_RagTag | Nuclear chromosome | Chromosome XI (S288C) | 330 | Correctly anchored |
| BK006945.2_RagTag | Nuclear chromosome | Chromosome XII (S288C) | 490 | Correctly anchored |
| BK006946.2_RagTag | Nuclear chromosome | Chromosome XIII (S288C) | 370 | Correctly anchored |
| BK006947.3_RagTag | Nuclear chromosome | Chromosome XIV (S288C) | 310 | Correctly anchored |
| BK006948.2_RagTag | Nuclear chromosome | Chromosome XV (S288C) | 380 | Correctly anchored |
| BK006949.2_RagTag | Nuclear chromosome | Chromosome XVI (S288C) | 260 | Correctly anchored |
| contig_29_pilon | Plasmid | 100% identity with J01347.1 | 27 | Complete 2μm plasmid sequence |
| contig_30_pilon | Plasmid (fragment) | Similar to J01347.1 | 14 | Duplicated plasmid fragment |
| contig_38_pilon | Plasmid (fragment) | Similar to J01347.1 | 10 | Another plasmid fragment |
| contig_2_pilon | Mitochondrial | 100% identity with OX377598.1 | 35 | Complete mitochondrial genome |
| contig_3_pilon | Mitochondrial (fragment) | 96.9% identity with CP006483.1 | 12 | Mitochondrial fragment |
| contig_36_pilon | Potentially unassigned nuclear | Chromosomes I and VI (in other strains) | 18 | Structurally variable region absent in S288C |
| contig_39_pilon | Potentially unassigned nuclear | Matches Chromosomes XI and XIV | 15 | Variable region or low coverage in the assembly |

**Supplementary Table 6.** Gene organization of contig_36 identified using AUGUSTUS gene prediction. Arrows indicate the orientation and relative position of the seven predicted coding sequences. Comparative analysis against the *S. cerevisiae* pangenome showed that all predicted genes correspond to conserved loci previously described in other strains.

| **Gene ID** | **Contig** | **Start** | **End** | **Strand** | **Length (aa)** | **Best hit (pangenome)** | **S288c ortholog** | **% Identity** | **Query coverage (%)** | **E-value** | **Interpretation** |
| --- | --- | --- | --- | --- | --- | --- | --- | --- | --- | --- | --- |
| g544.t1 | contig_36 | 8889 | 9578 | + | 230 | 48-MPR1-R | YFL058W/ THI5 | 99.7 | 100 | 0.0 | Conserved gene |
| g545.t1 | contig_36 | 10847 | 11869 | + | 341 | YFL058W | YFL058W/ THI5 | 96.6 | 99 | 0.0 | Conserved gene |
| g546.t1 | contig_36 | 12220 | 13239 | - | 339 | YFL056C | YFL056C/ AAD6 | 88.7 | 99 | 0.0 | Conserved gene |
| g547.t1 | contig_36 | 14847 | 16202 | + | 451 | YFL055W | YFL055W/ AGP3 | 98.9 | 99 | 0.0 | Conserved gene |
| g548.t1 | contig_36 | 18707 | 20647 | - | 646 | YFL054C | YFL054C/ AQY3 | 98.9 | 99 | 0.0 | Conserved gene |
| g549.t1 | contig_36 | 21278 | 23053 | + | 591 | YFL053W | YFL053W/ DAK2 | 99.8 | 99 | 0.0 | Conserved gene |
| g550.t1 | contig_36 | 26102 | 27499 | + | 412 | YFL052W | YFL052W/ ZNF1 | 99.7 | 99 | 0.0 | Conserved gene |

**Supplementary Table 7.** Statistical validation of growth parameter comparisons under thermal and osmotic stress conditions. Homogeneity of variance was assessed using Levene's test and residual normality using the Shapiro–Wilk test. Robustness of statistical inference was evaluated using one-way ANOVA, Welch's ANOVA, and permutation-based ANOVA. Agreement indicates concordance among the three statistical approaches. The only departure from residual normality was observed for lag phase at 38 °C; however, all statistical methods yielded the same conclusion.

| **Parameter** | **Assay** | **Condition** | **ANOVA p** | **Welch p** | **Permutation p** | **Levene p** | **Shapiro-Wilk p** | **Agreement** |
| --- | --- | --- | --- | --- | --- | --- | --- | --- |
| **μmax** | **Osmotic** | **0 M** | **<0.001** | **<0.001** | **0.003** | **0.247** | **0.555** | **Yes** |
| **μmax** | **Osmotic** | **1 M** | **<0.001** | **<0.001** | **0.003** | **0.735** | **0.274** | **Yes** |
| **μmax** | **Osmotic** | **1.5 M** | **<0.001** | **<0.001** | **0.003** | **0.414** | **0.762** | **Yes** |
| **μmax** | **Thermal** | **30 °C** | **<0.001** | **<0.001** | **0.004** | **0.247** | **0.555** | **Yes** |
| **μmax** | **Thermal** | **35 °C** | **<0.001** | **0.003** | **0.004** | **0.436** | **0.366** | **Yes** |
| **μmax** | **Thermal** | **38 °C** | **<0.001** | **<0.001** | **0.003** | **0.933** | **0.348** | **Yes** |
| **AUC** | **Osmotic** | **0 M** | **0.002** | **0.007** | **0.004** | **0.592** | **0.478** | **Yes** |
| **AUC** | **Osmotic** | **1 M** | **<0.001** | **<0.001** | **0.002** | **0.737** | **0.184** | **Yes** |
| **AUC** | **Osmotic** | **1.5 M** | **<0.001** | **<0.001** | **0.004** | **0.903** | **0.469** | **Yes** |
| **AUC** | **Thermal** | **30 °C** | **0.002** | **0.007** | **0.004** | **0.592** | **0.478** | **Yes** |
| **AUC** | **Thermal** | **35 °C** | **<0.001** | **<0.001** | **0.009** | **0.824** | **0.308** | **Yes** |
| **AUC** | **Thermal** | **38 °C** | **<0.001** | **<0.001** | **0.005** | **0.675** | **0.688** | **Yes** |
| **Lag** | **Osmotic** | **0 M** | **<0.001** | **0.002** | **0.013** | **0.581** | **0.860** | **Yes** |
| **Lag** | **Osmotic** | **1 M** | **<0.001** | **<0.001** | **0.004** | **0.503** | **0.963** | **Yes** |
| **Lag** | **Osmotic** | **1.5 M** | **<0.001** | **0.023** | **0.004** | **0.382** | **0.417** | **Yes** |
| **Lag** | **Thermal** | **30 °C** | **<0.001** | **0.002** | **0.013** | **0.581** | **0.860** | **Yes** |
| **Lag** | **Thermal** | **35 °C** | **<0.001** | **<0.001** | **0.004** | **0.759** | **0.236** | **Yes** |
| **Lag** | **Thermal** | **38 °C** | **0.414** | **0.144** | **0.427** | **0.129** | **0.044** | **Yes** |

**Supplementary Table 8.** Chilean strains included in the phylogenetic analysis. All strains belong to the Wine/European clade and originate from wine-related environments in Chile, as reported in Peter et al. (2018).

| **Standardized name** | **Isolation source** | **Ecological origin** | **Geographical origin** | **Clade** |
| --- | --- | --- | --- | --- |
| GAP | Wine | Wine | Chile | Wine/European |
| BTN | Wine | Wine | Chile | Wine/European |
| BTL | Wine | Wine | Chile | Wine/European |
| CRH | Wine | Wine | Chile | Wine/European |
| AGV | Grape juice | Wine | Chile | Wine/European |
| CRG | Wine | Wine | Chile | Wine/European |
